# Sensor Enabled Catheter Ablation Study (SECAS)

**DOI:** 10.7759/cureus.19550

**Published:** 2021-11-13

**Authors:** Hussein Rabah, Mahmoud Youness, Ali Rabah

**Affiliations:** 1 Department of Internal Medicine, Northwell Health, New York, USA; 2 Department of Internal Medicine, Faculty of Medical Sciences, Lebanese University, Beirut, LBN; 3 Division of Electrophysiology, Beirut Cardiac Institute, Beirut, LBN

**Keywords:** ensite precision, tachyarrhythmias, cardiac mapping, ablation, sensor enabled catheter

## Abstract

Background

EnSite Precision technology (Abbott, Chicago, Illinois) is a novel mapping and navigation system facilitating the visualization and manipulation of intracardiac catheters during arrhythmia ablation procedures. When using Sensor Enabled (SE) catheters (Abbott, Chicago, Illinois), the mapping system uses both electrical impedance and magnetic data to facilitate more accurate mapping and navigation. Whether this translates into better clinical outcomes is unknown.

Methods

This retrospective study will examine whether SE catheters improve the success rate or decrease the risks compared to Biosense Thermocool catheters (Biosense Webster Inc., Irvine, California) not employing sensor-enabled technology utilizing NavX EnSite Precision algorithms. Charts of 146 patients who underwent radiofrequency ablations for supraventricular and ventricular arrhythmias between 2016 and 2019 in the Beirut Cardiac Institute were reviewed and analyzed. It was concluded that SE catheters have the same success rate as electrical impedance catheters.

Results

A total of 70% of the ablations carried using the impedance-based catheter were successful compared to 74% using the SE catheter. However, the difference was statistically non-significant (p-value: 0.7). As for complications, the ventricular fibrillation rate was increased in the SE catheter group. Three procedures were complicated by pericardial effusion, three patients had reversible heart block, and one death was recorded, all reported while using the standard catheter (p-value: 0.01).

Conclusion

SE catheters have the same success rates compared to standard catheters using the EnSite Precision mapping system.

## Introduction

Radiofrequency catheter ablation (RFCA) has become an essential modality in treating patients with various types of arrhythmias [1]. The main indications for cardiac catheter ablation are recurrent/refractory arrhythmias or when medical treatment is intolerable or contraindicated. During the procedure, an electrophysiology study is conducted. The heart is mapped to measure its electrical activity and identify the temporal and spatial distributions of the electric potentials generated by the myocardium [2].

One of the widely used mapping systems is EnSite Precision technology (Abbott, Chicago, Illinois). It is a novel electroanatomical mapping and navigation system facilitating the visualization and manipulation of intracardiac catheters in any heart chamber for diagnostic and therapeutic applications. Abbott promised better-constructed maps and greater precision by introducing a Sensor Enabled (SE) catheter (Abbott, Chicago, Illinois) and applying the NavX SE field scaling [3]. By implementing magnet-based impedance distortion correction algorithms, maps created by SE catheters should be more accurate. Whether this translates into better clinical outcomes is unknown. This retrospective study examined whether SE catheters improve the success rate or decrease the risks compared to Biosense Thermocool catheters (Biosense Webster Inc., Irvine, California) utilizing electrical impedance (Eim) only, applied to the EnSite Precision system.

## Materials and methods

Methods

This is a retrospective study of 146 patients who underwent radiofrequency ablations for supraventricular and ventricular arrhythmias between 2016 and 2019 in the Beirut Cardiac Institute. IRB approval was obtained from the Beirut Cardiac Institute IRB committee, and information extracted from the medical records included age at the time of ablation, gender, indication for catheter ablation, type of catheter (Sensor Enabled by Abbott or electrical impedance only/Biosense Thermocool catheters), duration of the procedure, complications if any, and the outcome.

Patients were then distributed among two groups: group A includes patients who underwent the procedure using electrical impedance (Eim) catheters and group B includes patients who underwent the procedure using SE catheters.

Successful endpoints were defined as isolation of pulmonary veins in atrial fibrillation during ablation, termination of flutter with proof of bidirectional block across the isthmus, and non-inducible arrhythmia during an electrophysiology study during ventricular tachycardia (VT) ablation.

Statistics

Data analysis was performed using SPSS version 24.0 (IBM Corp, Armonk, NY). Continuous and categorical variables were presented as mean ± standard deviation and frequency/percentages, respectively. Pearson correlation coefficient, chi-square test, Fischer's exact test, t-test, and Mann-Whitney test were used for bivariate analyses. Graphical plots were used to evaluate the normality of continuous variables. Linear and binary logistic regression models were fitted to evaluate the effects of the catheter used, age, and sex on procedure success, operative time, and complications. Tests were interpreted below a significance level alpha = 0.05.

## Results

Studied population

The age of the patients at the time of the procedure ranged between 11 and 82 years. The mean age was 48 ± 17.8 years, with a median of 50 years. A total of 47 (32%) patients were females, and 99 (67.8%) were males.

Supraventricular tachycardia (SVT) ablation was the indication for 35 (24%) procedures. Patients included in this group were diagnosed with atypical atrial flutter or atrial fibrillation. A total of 111 (76%) ablations were indicated for VT. This group includes patients with ischemic and non-ischemic VT. The mean age of patients with supraventricular ablations was 43 years and 49.8 for patients with ventricular ablations (p-value: 0.04), with a mean duration of 199.4 minutes and 221.3 minutes, respectively (p-value: 0.2).

Catheter types and outcomes

The success rate of SVT ablation was 80% and that of the VT was 67.6%. Out of the 146 catheters used, 123 (84.2%) were standard, and 23 (15.8%) were SE catheters. A total of 103 (70.5%) procedures were successful, and 43 (29.5%) failed to ablate the arrhythmia. A total of 70% of the ablations carried using the impedance-based catheter were successful compared to 74% using the SE catheter. However, the difference was statistically non-significant (p-value: 0.7) (Table 1).

**Table 1:** The outcome of the ablation based on the catheter type. Eim: electrical Impedance; SE: Sensor Enabled.

		Successful	Unsuccessful	Total (N)	P-value
Catheter	Eim	86 (70%)	37 (30%)	123	
	SE	17 (74%)	6 (26%)	23	0.7
Total		103(70.5%)	43 (29.5%)	146	

Complications

During the procedures, three patients within each group had ventricular fibrillation (VF) requiring cardioversion. VF rate was increased in the SE catheter group. Three procedures were complicated by pericardial effusion, three patients had reversible heart block, and one death was recorded, all reported while using the Eim catheter (p-value: 0.01) (Table 2).

**Table 2:** The complications during the ablation procedures according to the type of catheter. Eim: electrical impedance; SE: Sensor Enabled; VF: ventricular fibrillation.

	Complications		None	VF	Effusion	Heart block			Death	TOTAL
Catheter	Eim	114 (92.7%)		3 (2.4%)	2 (1.6%)	3 (2.4%)		1 (0.8%)		123 (100%)
	SE	20 (87%)		3 (13%)	0 (0%)	0 (0%)	0 (0%)			23 (100%)
TOTAL		134 (91.8%)		6 (4.1%)	2 (1.4%)	3 (2.1%)	1 (0.7%)			146 (100%)

## Discussion

Since its introduction in the early 1990s, more complex arrhythmias are being treated using radiofrequency ablation. This requires precise navigation and representation of the ablation catheter site within the heart and combining both spatial and temporal data to identify the arrhythmogenic focus. Therefore, cardiac mapping is a crucial step during this procedure. Traditionally, catheter navigation was performed under fluoroscopy guidance [4]; however, this requires the administration of ionizing radiation, which carries a risk for both patients and staff [5].

In recent years, the EnSite NavX mapping system was introduced. It facilitates non-fluoroscopic three-dimensional electroanatomic mapping while maintaining accurate navigation [6]. Fernández-Gómez et al. reported that the EnSite NavX application eliminates the need for fluoroscopy in 94.7% of right-sided SVT cases [7]. During the same year, Stec et al. concluded that EnSite NavX system navigation resulted in complete elimination of fluoroscopy and the use of protective lead aprons by the electrophysiology staff in approximately 95% of SVT ablations with no changes in success rates of the procedures or adverse event frequencies [8].

The EnSite NavX navigation system uses a constant current over three pairs of nominally orthogonal patches on the patient to create an impedance gradient across the thorax. The orthogonal patches are placed on the patient's side, chest, and back, and the back of the neck and inner left thigh, creating the x, y, and z-axis, respectively. The three patch pairs send three independent, low-power currents through the patient's chest in three orthogonal (x, y, and z) directions. When an electrode is maneuvered within the established impedance gradient, NavX measures the local impedance in the respective plane and calculates its position along multiple axes. This creates a three-dimensional electrical navigation field that allows exact localization of the catheter within the cardiac chambers. The system saves the coordinates of each electrode relative to a positional reference, which is a patch placed on the patient's abdomen.

On the other hand, the EnSite Precision is a three-dimensional navigation system that integrates electrical impedance and magnetic field data. As in the EnSite NavX, three orthogonal electrode pairs form three orthogonal axes (x-y-z) across the chest. Two patient reference sensors (PRS) are also attached to the patient's chest and back and function as sensors for metal distortion and movement.

When the surface electrodes are connected to the EnSite Precision system, a transthoracic electrical field is created by sending 8-kHz alternate signals through each of the three patches pairs. As the catheter is advanced into the transthoracic field, each catheter electrode senses voltage, which is then adjusted to the voltage gradient on all three axes. This creates a three-dimensional model of the heart and allows real-time navigation through detecting multiple catheter electrodes simultaneously.

In addition, EnSite Precision increases the accuracy by less than 1 mm using magnetic field localization technology. A low-power magnetic field is generated using a field frame, within which the SE catheter can be localized. When a SE catheter is introduced into the navigation system, SE field scaling is applied. The EnSite Precision system then integrates electrical impedance and magnetic data to create an optimal navigation field [9].

This is the first study to describe the clinical outcomes of SE catheters compared to Eim catheters using the EnSite Precision mapping system. Out of the 146 patients enrolled in the study, 24% had supraventricular arrhythmias, while 76% had ventricular tachycardias. A total of 123 (84.2%) of the catheters used were conventional, while 23 (15.8%) were SE catheters.

Overall, 70.5% of the ablations were successful. However, there was no statistically significant difference between the success rate of SE catheters compared to Eim catheters (p-value: 0.7). This shows that SE catheters do not have a higher success rate than Eim catheters when utilizing EnSite Precision.

Regarding the complications, the SE catheter group had a higher rate of ventricular arrhythmias requiring electrical cardioversion (13% versus 2.4%). Those complications were probably a result of ventricular arrhythmia induction during the procedure and therefore do not reflect catheter-type-related complications. On the other hand, Eim's use was associated with a higher rate of transient heart block (2.4% versus 0%) and pericardial effusion (1.6% versus 0%). Those complications might be related to the catheter type, where the SE catheters might provide better accuracy and more delineation and therefore could decrease the risk of cardiac conduction system damage and myocardial perforation (Table 2). However, such results cannot be concluded due to the limitations of the sample size.

Study limitations

A primary limitation of the present study is the small sample size of the SE catheter group. As there are no reports till this date of studies comparing SE catheters to the Eim catheters, we could not assess the power of our groups, neither did we have access to more SE catheters for logistic reasons; as a result, both catheter groups were not allocated equally and hence underpower is possible. We have not reduced the size of the control group to reach the statistically determined sample size. Therefore, future investigations with larger samples are needed to compare with our findings.

## Conclusions

SE catheters have the same success rates compared to standard catheters using the EnSite Precision mapping system. However, SE catheters were associated with a lower rate of transient conduction blocks. Further prospective studies should be conducted regarding this matter.

## References

[1] Miller JM, Zipes DP (2002). Catheter ablation of arrhythmias. Circulation.

[2] Ladas TP, Sugrue A, Nan J, Vaidya VR, Padmanabhan D, Venkatachalam KL, Asirvatham SJ (2019). Fundamentals of cardiac mapping. Card Electrophysiol Clin.

[3] EnSite Precision cardiac mapping system. https://www.cardiovascular.abbott/us/en/hcp/products/electrophysiology/mapping-systems/ensite.html.

[4] Papagiannis J, Beissel DJ, Krause U (2017). Atrioventricular nodal reentrant tachycardia in patients with congenital heart disease: outcome after catheter ablation. Circ Arrhythm Electrophysiol.

[5] Durán A, Hian SK, Miller DL, Le Heron J, Padovani R, Vano E (2013). A summary of recommendations for occupational radiation protection in interventional cardiology. Catheter Cardiovasc Interv.

[6] LaPage MJ, Saul JP (2011). Update on rhythm mapping and catheter navigation. Curr Opin Cardiol.

[7] Fernández-Gómez JM, Moriña-Vázquez P, Morales Edel R, Venegas-Gamero J, Barba-Pichardo R, Carranza MH (2014). Exclusion of fluoroscopy use in catheter ablation procedures: six years of experience at a single center. J Cardiovasc Electrophysiol.

[8] Stec S, Śledź J, Mazij M (2014). Feasibility of implementation of a "simplified, no-X-ray, no-lead apron, two-catheter approach" for ablation of supraventricular arrhythmias in children and adults. J Cardiovasc Electrophysiol.

[9] Borlich M, Sommer P (2019). Cardiac mapping systems: Rhythmia, Topera, EnSite Precision, and CARTO. Advances in Cardiac Mapping and Catheter Ablation: Part I, An Issue of Cardiac Electrophysiology Clinics.

